# Superior Mesenteric Artery originating from the celiac axis: A rare vascular anomaly

**DOI:** 10.1186/1477-7819-9-71

**Published:** 2011-07-12

**Authors:** Michael G Wayne, Rahul Narang, Suzanne Verzosa, Avram Cooperman

**Affiliations:** 1 Pancreas Center at Beth Israel Medical Center, NY 37 Union Square West, 4th floor, NY 10003, USA

## Abstract

The knowledge of the vascular anatomy of the concerned region is an important prerequisite for planning surgical intervention. The awareness of the existing vascular anomalies enhances the insight regarding that region. We report a patient undergoing preoperative evaluation with CTA finding of Superior Mesenteric Artery (SMA) originating from the celiac artery. This celiac-mesenteric trunk is rare (<1%).

## Case Presentation

A 74-year-old woman was referred by her gastroenterologist with painless jaundice. She presented with several months of decreased appetite and a three week history of light colored stool with dark urine. An endoscopic ultrasound was performed and revealed a hypoechoic, irregular, 3.4 cm mass in the head of the pancreas. The common bile duct and pancreatic duct were obstructed from the mass. No vascular invasion, celiac or peri-celiac lymph nodes were noted. Two biliary stents were placed and no biopsies were taken during the procedure.

**Figure 1:**
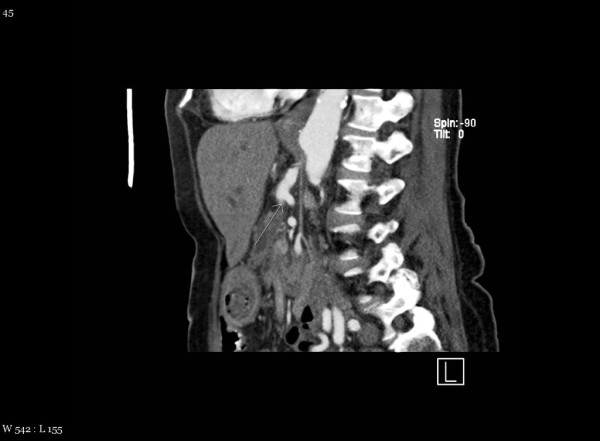
**CT demonstrating abnormal celiac origin**.

**Figure 2:**
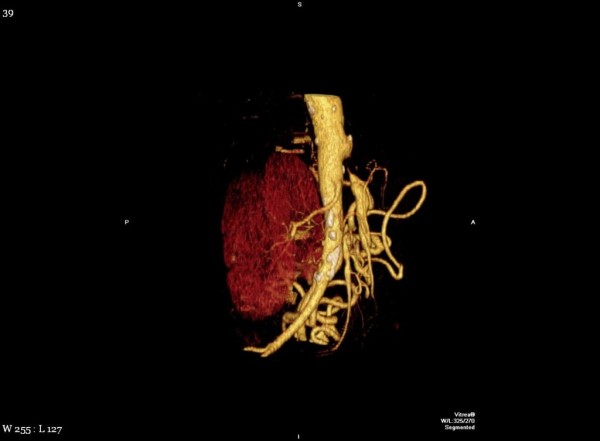
**CT reconstruction showing vascular anomaly**.

**Figure 3:**
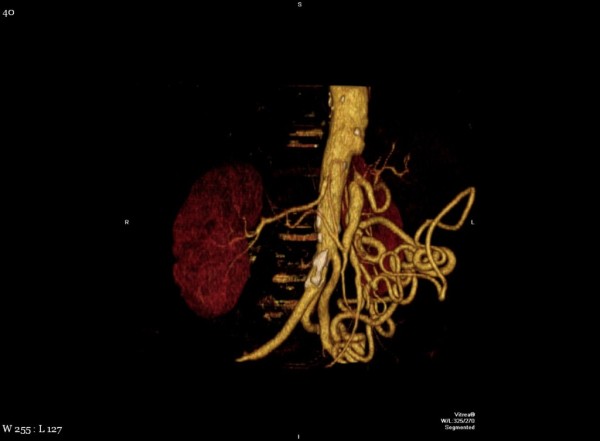
**CT reconstruction showing vascular anomaly**.

Prior to considering the patient a candidate for surgery, a high resolution computed tomography (CT) scan was performed with pancreatic protocol in non-contrast, arterial and venous phase to determine resectablity. CT scan was consistent with a double duct sign with markedly dilated pancreatic and common bile duct and intrahepatic biliary dilation secondary to mass on the pancreatic head. An interesting variant in anatomy was also identified, which was important for proper surgical planning. The superior mesenteric artery was found to be originating from the celiac axis. (Figure 1, 2, 3)

Pancreaticoduodenectomy is utilized selectively in the management of patients with neoplastic lesions of the pancreas and periampullary region. In these patients, the role of CT angiography (CTA) is important in determining tumor respectability and it allows one to evaluate for variant arterial anatomy. Preoperative knowledge of variant anatomy can assist in selection of treatment options and facilitate in surgical dissection and avoid iatrogenic injury.

The celiac artery supplies the liver, spleen, pancreas, and some of the stomach and duodenum. The superior mesenteric artery (SMA) supplies the small intestine, ascending colon, and a large portion of the transverse colon. Variation of arterial anatomy is common and occurs in nearly half of the population [1]).

We report a patient undergoing preoperative evaluation with CTA finding of Superior Mesenteric Artery (SMA) originating from the celiac artery. This celiac-mesenteric trunk is rare (<1%), however has been described [2].

In the embryo, the three paired arteries of the trunk originate from the aorta. Posterior arteries are parietal, lateral arteries are urogenital, and anterior arteries are intestinal. In human embryos the primitive intestinal arteries (vitelline arteries) are connected by a Tandler's anterior longitudinal anastomosis [3]. When the connection between celiac trunk and SMA remains presents, it tends to form a small vertical arch just behind the body of the pancreas. The rarely reported arterial anastomosis between the celiac trunk and SMA is known as the arc of Bühler's according to McNulty et al. [4]. An arc of Bühler was identified in 4 patients (3.3%) out of 120 combined celiac and superior mesenteric artery angiograms, in a study by Saad et al. [5]. In one study the arc of Bühler was identified in 14 cases among 340 selective celiac and superior mesenteric arteriographic studies [6]. They also stated that the arc of Bühler between the celiac and superior mesenteric arteries has to be considered as an embryological persistence 10th and 13th primitive arteries, which is associated with the persistence of ventral longitudinal anastomosis [2,6]).

In our patient the CTA also demonstrated a subtotal occlusion of the origin of the celiac axis. There was significantly enlarged inferior mesenteric artery, which is likely due to the retrograde perfusion of SMA and celiac arteries.

## Conclusion

It is important to understand the vascular anatomy of a region in planning a surgical intervention. When performing a pancreaticoduodenectomy, an awareness of the vasculature is necessary in case vasculature reconstruction needs to be performed because of tumor involvement of the vessels. Knowing the existing vascular anomalies enhances the insight regarding that area and helps to prevent mistakes due to a lack of awareness. Our patient underwent a pancreaticoduodenectomy. There was no vessel involvement found during the case. The patient tolerated the procedure well and was discharged in a timely fashion, without complication.

## Consent

Written informed consent was obtained from the patient for publication of this Case report and any accompanying images. A copy of the written consent is available for review by the Editor-in-Chief of this journal.

## Competing interests

The authors declare that they have no competing interests.

## Authors' contributions

MW-lead author and primary surgeon for the patient. RN-assisted in writing the paper. SV-gathered and edited all the images. AC-assistant surgeon and edited the final paper. All authors read and approved the final manuscript.
